# Evaluation of organ doses and effective dose according to the ICRP Publication 110 reference male/female phantom and the modified ImPACT CT patient dosimetry

**DOI:** 10.1120/jacmp.v15i5.4823

**Published:** 2014-09-07

**Authors:** Masanao Kobayashi, Yasuki Asada, Kosuke Matsubara, Yuta Matsunaga, Ai Kawaguchi, Kazuhiro Katada, Hiroshi Toyama, Kichiro Koshida, Shouichi Suzuki

**Affiliations:** ^1^ Department of Radiology Fujita Health University Hospital Tokoake Japan; ^2^ Graduate School of Medical Sciences Division of Medical Sciences, Kanazawa University Kanazawa Japan; ^3^ Graduate School of Health Sciences Fujita Health University Toyoake Japan; ^4^ Department of Imaging Nagoya Kyoritsu Hospital Nagoya Japan; ^5^ Department of Radiology Toyota Memorial Hospital Toyota Japan; ^6^ Department of Radiology Fujita Health University School of Medicine Toyoake Japan

**Keywords:** computed tomography, ImPACT, effective dose, organ dose, ICRP

## Abstract

We modified the Imaging Performance Assessment of CT scanners (ImPACT) to evaluate the organ doses and the effective dose based on the International Commission on Radiological Protection (ICRP) Publication 110 reference male/female phantom with the Aquilion ONE ViSION Edition scanner. To select the new CT scanner, the measurement results of the ${\text{CTDI}}_{100,c}$ and ${\text{CTDI}}_{100,p}$ for the 160 (head) and the 320 (body) mm polymethylmethacrylate phantoms, respectively, were entered on the Excel worksheet. To compute the organ doses and effective dose of the ICRP reference male/female phantom, the conversion factors obtained by comparison between the organ doses of different types of phantom were applied. The organ doses and the effective dose were almost identical for the ICRP reference male/female and modified ImPACT. The results of this study showed that, with the dose assessment of the ImPACT, the difference in sex influences only testes and ovaries. Because the MIRD‐5 phantom represents a partially hermaphrodite adult, the phantom has the dimensions of the male reference man including testes, ovaries, and uterus but no female breasts, whereas the ICRP male/female phantom includes whole‐body male and female anatomies based on high‐resolution anatomical datasets. The conversion factors can be used to estimate the doses of a male and a female accurately, and efficient dose assessment can be performed with the modified ImPACT.

PACS number: 87.53.LY, 87.57.Q‐, 87.57.‐s

## I. INTRODUCTION

A recent survey carried out by the Institute of Regional Studies at Ibaraki University has found that the number of people concerned about radiation exposure, including that from medical X‐ray examinations, has increased since the Fukushima Daiich nuclear plant in Japan was affected by the earthquake and tsunami natural disasters.^1^, ^2^, ^3^ Of note, Japan has the highest annual frequency of diagnostic X‐rays, and nearly half of the total medical radiation exposure is from X‐ray computed tomography (CT).^4^, ^5^, ^6^ Therefore, it is of interest to estimate the radiation dose in CT.

The effective dose, which is useful for comparing examinations with different techniques such as radiography, CT, and nuclear medicine, is currently deemed the best available dose descriptor for quantifying stochastic risks in diagnostic radiology. Hence, it is not applicable to any single individual. To obtain the effective dose in CT, the effective dose per unit dose length product (DLP) conversion factor is required, also known as the k‐factor in the International Commission on Radiological Protection (ICRP) Publication 102.^7^, ^8^, ^9^, ^10^ The applicable DLP is displayed on the CT console at the end of the procedure and can be used to quantify the total amount of radiation received during any given scan.^(11)^ Although the k‐factors are practical, they are based on data averaged over many scanner makes and models and are, therefore, not specific to a selected scanner.

The Imaging Performance Assessment of CT scanners (ImPACT) group, the Scanner Evaluation Center of the United Kingdom National Health Service, developed an Excel (Microsoft) spreadsheet to provide a convenient user interface for determining organ doses by using the National Radiological Protection Board (NRPB) Monte Carlo dose datasets (i.e., NRPB‐SR250).^12^, ^13^ The ImPACT CT patient dosimetry version 1.04 (released in May 2011) reflects further development of a method to map results from the original 23 scanner datasets to new CT scanners by applying the so‐called “ImPACT factors.” These factors are based on tube voltage‐dependent CT dose index free‐in‐air (${\text{CTDI}}_{\text{air}}$) and CTDI in the center (${\text{CTDI}}_{100,c}$) with either a standard head or standard body polymethylmethacrylate (PMMA) phantom. For the mathematical phantom, the medical international radiation dose‐five (MIRD‐5) phantom was divided from head to mid‐thigh into 208 axial slabs of 5 mm thickness.^14^, ^15^ Then, accounting for the scan condition and using the CT scanner‐specific data for geometry and beam shaping, they simulated a CT scan and calculated the organ doses for the irradiation of each axial slab. Hence, this method is validated for bolus tracking, test injection, and dynamic scan. However, use of such software always requires the above‐mentioned new basic data. To date, little has been reported on novel usage of the ImPACT. We modified the ImPACT to evaluate the organ doses and the effective dose based on the ICRP Publication 110 reference male/female (ICRP male/female) phantom by using the conversion factors for the Aquilion ONE ViSION Edition scanner.^(16)^

## II. MATERIALS AND METHODS

### A. Measurement of the CT dose index

A multidetector row CT scanner with 320 rows of detector elements (320‐MDCT; Aquilion ONE ViSION Edition; Toshiba Medical Systems, Otawara, Japan), which is capable of data acquisition at a slice thickness of 0.5 mm with a coverage of 160 mm, was used in this study. For evaluation of the CTDI, a pencil chamber with an effective length of 100 mm and a volume of 3 cm^3^ (3CT, Radcal Corporation, Monrovia, CA) was used. The formula for calculation of the ${\text{CTDI}}_{100}$ is shown below:

(1) $$CTDI_{100}=\int_{-50mm}^{+50mm}\frac{D(z)}{\text{min}\{NT,100\}}dz$$

where *N, T*, and *D(z)* indicate the number of detector rows, nominal slice thickness, and dose distribution along the z‐axis, respectively. When NT is greater than 100 mm, the physical meaning of the ${\text{CTDI}}_{100}$ changes from the average dose at the center of a 100 mm scan length to the average dose over the central 100 mm region of a single axial scan.^(17)^ The dose measurement result at the center of the PMMA phantom is defined as the ${\text{CTDI}}_{100,c}$ and the average dose of the peripheral locations at the 12, 3, 6, and 9 o'clock positions is defined as the ${\text{CTDI}}_{100,p}$.^7^, ^8^, ^11^, ^17^ Scan protocols were as follows: tube voltage was 80, 100, 120, or 135 kV; tube current was 200 mA; nominal beam width (i.e., collimation) was 16 mm; field‐of‐view was 320 mm; and X‐ray tube rotation time was 0.5 s. To obtain the relative CTDI (i.e., Rel. CTDI), the CTDI values at nominal beam widths of 2–160 mm, displayed on the CT console, were compared.

## B. Modification of the ImPACT CT patient dosimetry

The following procedure was used to temporarily set the security level to enable all macros: 1) On the developer tab, in the code group, macro security was clicked; 2) Under macro settings, enable all macros (not recommended, potentially dangerous code can run) was clicked; 3) To unlock any cells or spreadsheets, on the review tab, in the changes group, unprotect sheet was clicked. Then, the ImPACT CT patient dosimetry version 1.04 (ImPACT) was used in this study.

### B.1. Addition of CT scanner data

**Figure 1:**
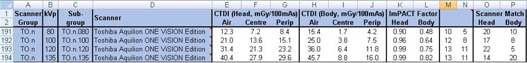
Setup of the CT scanner data on the Scanners worksheet. The CTDI data were inputted using the measured value per 100 mAs of the 160 (head) or the 320 (body) mm PMMA phantom. Then, the ImPACT factor, blank space, and scanner match were inputted automatically.

To select the new CT scanner (i.e., 320‐MDCT) on the ScanCalculation worksheet of the ImPACT, the Scanners worksheet was modified as follows: scanner group, “TO.n”; kVP, “80 to 135”; sub‐group, “TO.n.80 to 135”; scanner, “Toshiba Aquilion ONE ViSION Edition”; and CTDI, “measured value.” Then, ImPACT factor (ImF), blank space, and Scanner Match were input automatically (Fig. 1). For the ImPACT factor, a reasonable correlation is obtained with the effective dose and the X‐ray beam half‐value layer as follows:^(12)^

(2) $$Head:0.4738\times \frac{CTDI_{100,c}}{CTDI_{100,air}}+0.8045\times \frac{CTDI_{100,p}}{CTDI_{100,air}}+0.0752$$

(3) $$Body:3.5842\times \frac{CTDI_{100,c}}{CTDI_{100,air}}+0.6328\times \frac{CTDI_{100,p}}{CTDI_{100,air}}-0.0902$$

**Figure 2:**
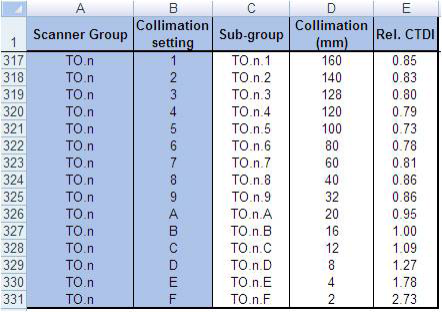
Setup of the CT collimation data on the Collimation worksheet. The Rel. CTDI was inputted by the correlation of collimation and the CTDI. To input the Rel. CTDI, the CTDI value displayed on the scanner console was observed. Then, the measured CTDI of slice thickness at 16 mm was normalized to 1.0.

For blank space and Scanner Match, the values were based on the MatchData worksheet. In addition, the Collimation worksheet was modified as follows: scanner group, “TO.n”; collimation setting, “1 to F”; sub‐group, “TO.n.1 to F”; and collimation, “160 to 2.” For Rel. CTDI, the correlation of collimation and the CTDI was inputted (Fig. 2). To use these data, on the ScanCalculation worksheet, in the combobox, input range of form setting was changed.

The organ doses and the effective dose were calculated for 320‐MDCT and Aquilion 16 (16‐MDCT) scanners at a tube voltage of 120 kV, tube current of 200 mA, and X‐ray tube rotation time of 0.5 s/rot, with nominal beam widths of 160 and 32 mm, a scan length of 160 mm, a pitch factor of 1.0, a scan region of the head, a start position of 78 mm, and an end position of 94 mm. For the scan mode, radiation exposure was simulated as contiguous axial scans to cover 160 mm using 32 mm nominal beam widths and as a single axial scan with a 160 mm (i.e., volume scan) nominal beam width (no table motion). Dose calculations were performed using the original MIRD‐5 phantom.

### B.2. Addition of conversion factors for the ICRP 110 phantom

**Figure 3:**
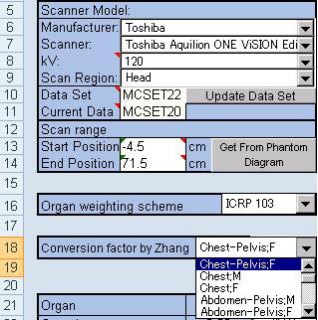
Setup of the combobox on the Scancalculation worksheet. Refer to Fig. 4 for the list.

**Figure 4:**
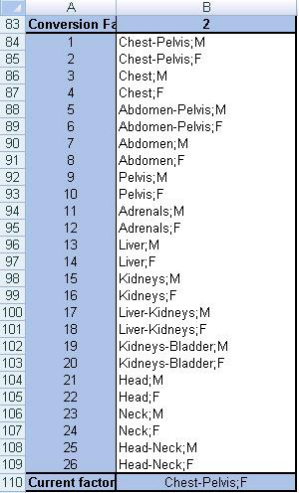
Setup of the 13 examination categories (chest‐abdomen‐pelvis, chest, abdomen‐pelvis, abdomen, pelvis, adrenals, liver, kidneys, liver‐kidneys, kidneys‐bladder, head, neck, and head‐neck) of the conversion factors for male (M)/female (F) on the Selections worksheet.

Modification of the ImPACT spreadsheet can be used to compute the organ doses and the effective dose in the ICRP 110 phantom by using the conversion factors, which were obtained by comparison between the organ doses of different types of phantom and reported by Zhang et al.^(18)^ The combobox was copied and pasted to select the conversion factors (Fig. 3). To select the conversion factors, the Selections worksheet was modified as follows: A83, “Conversion factor (CF)”; A84‐110, “1‐27”; A111, “Current factor”; B83 was first named as WF (i.e., Name Box is WF); B84‐110, “Thirteen examination categories (chest‐abdomen‐pelvis, chest, abdomen‐pelvis, abdomen, pelvis, adrenals, liver, kidneys, liver‐kidneys, kidneys‐bladder, head, neck, and head‐neck) of the conversion factors for male (M)/female (F)” and MIRD‐5; and B111,“=VLOOKUP(WF,A84:B109,2)” (Fig. 4). Then, the input range of form setting in the combobox was defined as “Selections!$B$84:$B$110.” In addition, the selected examination category was linked as “Selections!$B$83” (Fig. 4).

**Figure 5:**
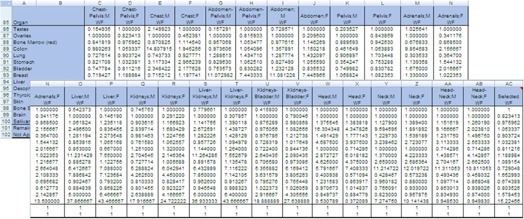
Setup of the CFs for the 13 categories. CFs were obtained from Fig. 4.

To estimate the organ doses and the effective dose, WFs were obtained as follows:

(4) $$CF=\frac{D_{T,ICRP110}}{D_{T,MIRD-5}}$$

where $D_{T}$ are organ and tissue doses simulated by the ICRP 110 and MIRD‐5, which have been reported by Zhang et al.^(18)^ CFs were tabulated (Fig. 5). To use these data, on the ScanCalculation worksheet, in the combobox, input range of form setting was changed. For the cells “AD86‐100”, CFs used in this dosimetry were detailed as “example line 86; =CHOOSE(CFs,C86,D86,E86, F86,G86,H86,I86,J86,K86,L86,M86,N86,O86,P86,Q86,R86,S86,T86,U86,V86,W86,X86,Y8 6,Z86,AA86,AB86,AC86).” The individual organ and tissue doses were then weighted by CFs and summed to obtain the effective dose.

(5) $$D_{T}=D_{T,MIRD-5}{\;}^{\times CF}$$

(6) $$H_{T}=D_{T}\times W_{R}$$

(7) $$ED=\sum_{T}H_{T}\times W_{T}$$

where $W_{R}$ is the radiate on the weighting factor, $H_{T}$ is the equivalent dose, and $W_{T}$ is the tissue weighting factor defined by ICRP publication 103.^(19)^

The organ and tissue doses were estimated for the general‐2 (chest‐abdomen‐pelvis and head) examination categories. The results were then compared with the organ doses for the ICRP 110, which have been reported by Zhang et al.^(18)^ These doses were obtained using the GE Healthcare LightSpeed VCT scanner (GE Healthcare, Waukesha, WI) at a tube voltage of 120 kV, tube current of 200 mA, and X‐ray tube rotation time of 0.5 s/rot. For the chest‐abdomen‐pelvis CT examination, the simulation pitch factor was 1.375, beam width was 40 mm, and simulated image coverage was lung apex (‐4.5cm) to inferior ischium (71.5 cm). For the head CT examination, the simulation pitch factor was 1.0, beam width was 20 mm, and simulated image coverage was vertex of skull (81 cm) to scalp bottom (94 cm). Dose calculations were performed using the conversion factors of “Chest‐Pelvis; M/F” and “Head; M/F,” respectively.

## III. RESULTS

### A. Comparison of 320‐MDCT and 16‐MDCT

**Figure 6:**
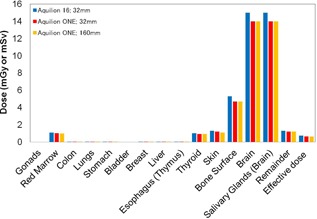
Comparison between different scanner types in terms of the organ doses and the effective dose of the head.

Estimation of the organ doses and effective dose (Fig. 6) of two types of CT scanners for the scan region of the head showed that the organ doses of 320‐MDCT were 1 mGy lower for the brain and salivary glands than those of 16‐MDCT. The difference between effective dose for 320‐MDCT (0.64 mSv) and 16‐MDCT (0.72 mSv) was not as dramatic as the differences in organ doses. In addition, the organ doses and effective dose were almost identical at the nominal slice thicknesses of 32 mm and 160 mm with 320‐MDCT.

### B. Comparison of dose of ICRP 110 and MIRD‐5

Estimation of the organ doses and the effective dose (7, 8) with the LightSpeed VCT scanner in the chest‐pelvis examination showed that most organ doses of the MIRD‐5 phantom were larger than those of the ICRP 110 male phantom estimated by Zhang.^(18)^ On the other hand, the breasts and thyroid doses of the MIRD‐5 were smaller than those of the ICRP 110 female.

Estimation of the organ doses and the effective dose (9, 10) in the head examination showed that the brain and salivary gland doses of the MIRD‐5 were larger than those of the ICRP 110. These results have been reported by Zhang.^(18)^

The organ doses and the effective dose were almost identical for the ICRP 110 and the modified ImPACT.

**Figure 7:**
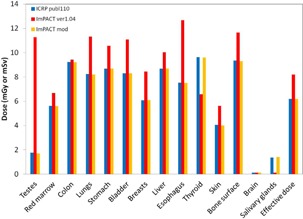
Comparison between different types of phantoms in terms of the organ doses and the effective dose in the chest‐abdomen of the male. Note that doses for ICRP 110 are reported by Zhang et al.(^18^)

**Figure 8:**
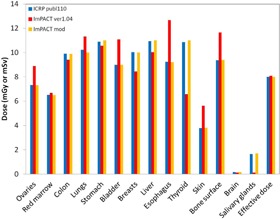
Comparison between different types of phantoms in terms of the organ doses and the effective dose in the chest‐abdomen of the female. Note that doses for ICRP 110 are reported by Zhang et al.(^18^)

**Figure 9:**
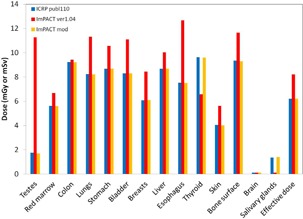
Comparison between different types of phantoms in terms of the organ doses and the effective dose in the head of the male. Note that doses for ICRP 110 are reported by Zhang et al.^(18)^

**Figure 10:**
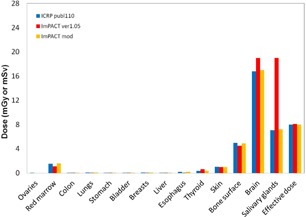
Comparison between different types of phantoms in terms of the organ doses and the effective dose in the head of the female. Note that doses for ICRP 110 are reported by Zhang et al.^(18)^

## IV. DISCUSSION

In this study, we modified the ImPACT to obtain the organ doses and the effective dose of 320‐MDCT based on the ICRP 110 phantom. To date, several CT studies have determined the organ doses and the effective dose by using the default CT scanner of the ImPACT. Considering the future, we explained in detail how to apply the basic data of the CT scanner and the conversion factors of the ICRP 110 phantom by using the conversion factors obtained by comparison between the organ doses of different types of phantom and reported by Zhang et al.^(18)^ Results of the organ doses and effective dose for 320‐MDCT and 16‐MDCT (Fig. 6), obtained using the ImPACT, showed that the doses were almost identical; this demonstrated that calculation results using the conversion factors for the ICRP 110 are reliable (7, 8, 9, 10).

The latest version of the ImPACT CT dosimetry, version 1.04, was released in 2011, and this software has not been updated for three years. Doses for the Toshiba Aquilion 64 (64‐MDCT) can be calculated using the 16‐MDCT specifications.^(12)^ For these scanners, specification of each scanner was reported by the Center for Evidence‐based Purchasing (CEP) report 08025 and report 08027.^20^, ^21^ These specifications of CT scanners provide data of parameters, such as detector array and filtration. It is reported that the total effective length of detector array (32 mm) and total filtration at max kV on central axis (L wedge filter, 2.5; M wedge filter, 4.0; and DR wedge filter, 11.0) are the same; therefore, 64‐MDCT and 16‐MDCT doses can be calculated in the same manner. However, 320‐MDCT has a 160 mm detector array, together with a 4.8L wedge filter, 4.8M wedge filter, and 3.9S wedge filter.^(22)^ In addition, 320‐MDCT has 320 rows of detector elements, which are capable of data acquisition at a slice thickness of 0.5 mm with a coverage of 160 mm. Therefore, 320‐MDCT dose formulas differ from those of 16‐MDCT. The data for the studied scanner were not reported by the ImPACT group; therefore, we performed the required scanner matching according to their method using measurements from our scanner. The results of this study showed that the estimations of organ doses and effective dose of 320‐MDCT with the ImPACT were almost identical to those of 16‐MDCT.^(12)^

An adaptation of this method was required for the single‐axial scan, because the input parameters of slice thickness and pitch factor used to compute the effective dose were fixed by the CT scanner. Thus, for the body examination, it was not appropriate to use the scanner model of 16‐MDCT.

The anthropomorphic models (also called mathematical phantoms) utilized for the dose calculations in the ImPACT, use stylized geometric definitions that were developed for reference adults. The models' exteriors comprise three sections: a truncated elliptic cylinder representing the head and neck, an elliptic cylinder representing the torso and hips, and a truncated elliptic cone representing the legs. Several organs and tissues are mathematically defined as occupying finite spaces within the body space.^(15)^ As an improvement to these models, the ICRP Task Group on Reference Man has developed a new type of anatomical phantom. ICRP 110 introduced the official computational models representing the adult reference male/female according to CT, magnetic resonance, and other images obtained from high‐resolution scans of a single individual.^(16)^ For the dose assessment, the organ or tissue doses are calculated by sex‐averaging of values obtained using reference male/female phantoms. Then, the effective dose is calculated using revised age‐ and sex‐averaged tissue weighting factors based on updated risk data, and intended to apply as rounded values to a population of both sexes and all ages. Hence, the effective dose is calculated for a reference person and not for an individual. These definitions have been introduced in the ICRP103.^(19)^ The actual doses of the reference male/female are required to estimate the effective dose with the ImPACT. The results of this study showed that, in the dose assessment of the ImPACT, the difference in sex influenced only testes and ovaries. Because the MIRD‐5 phantom represents a partially hermaphrodite adult, the phantom has the dimensions of the male reference man including testes, ovaries, and uterus, but no female breasts, whereas the ICRP 110 phantom includes whole‐body male and female anatomies based on high‐resolution anatomical datasets. The conversion factors can be used to estimate the dose of a male and a female.^(18)^ Therefore, ImPACT could be useful for evaluation of the organ doses and effective dose.

A potential weakness of this study is the inherent uncertainty of utilizing the CFs obtained for the GE Healthcare LightSpeed VCT scanner only; thus, influence of the effective energy has not been considered. Another limitation is that use of the CFs of the predefined scan protocols as listed is complicated and limits scan range selection flexibility.

Future advances include the autoexposure control (AEC) system and the electrocardiograph (ECG) gate scan, which warrant modification. AEC systems for CT scanners are now available for all the major scanners, and the National Institute of Radiological Sciences (NIRS) has indicated the directivity of the usage of AEC by WAZA‐ARI, which is a Web‐based radiation‐exposure CT examination system derived by Monte Carlo calculations using the Particle and Heavy Ion Transport code System (PHITS).^23^, ^24^, ^25^, ^26^ For the ECG gate scan, studies are in progress to evaluate coronary‐artery CT. These applications need to be taken into consideration by the ImPACT.

## V. CONCLUSIONS

This study showed that novel and efficient dose assessment was able to be performed with the modified ImPACT, which may be useful in future applications.

## ACKNOWLEDGMENTS

We especially thank Dr. Yakun Zhang for supplying the data. In addition, we would like to thank Dr. Alan Britten for permission to use of the ImPACT.

## Supporting information

Supplementary Material

Supplementary Material
