# Supplementary material for: Evaluation of organ doses and effective dose according to the ICRP Publication 110 reference male/female phantom and the modified ImPACT CT patient dosimetry
Source: J Appl Clin Med Phys. 2014 Sep 7;15(5):246–56. doi: 10.1120/jacmp.v15i5.4823 (PMC5711082; doi:10.1120/jacmp.v15i5.4823)
Supplement: Supplementary file 1 — Supplementary Material [file ACM2-15-246-s001.docx]

Evaluation of organ doses and effective dose according to the ICRP publication 110 reference male/female phantom and the modified ImPACT CT patient dosimetry

**Masanao Kobayashi,^1,2^, Kichiro Koshida,^2^ Shoichi Suzuki,^3^ Kosuke Matsubara^2^, Yuta Matsunaga,^4^ Ai Kawaguchi,^5^ Kazuhiro Katada,^6^**

*Department of Radiology^1^, Fujita Health University Hospital, Tokoake, Japan; Kanazawa University^2^, Graduate School of Medical Sciences, Division of Medical Sciences, Kanazawa, Japan; Graduate School of Health Sciences^3^, Fujita Health University, Toyoake, Japan; Department of Imaging^4^, Nagoya Kyoritsu Hospital, Nagoya, Japan; Department of Radiology^5^, Toyota Memorial Hospital, Toyota, Japan; Department of Radiology^6^, Fujita Health University School of Medicine, Toyoake, Japan*

[*masa1121@fujita-hu.ac.jp*](mailto:masa1121@fujita-hu.ac.jp)

Evaluation of dose with the modified ImPACT

Evaluation of organ doses and effective dose according to the ICRP publication 110 reference male/female phantom and the modified ImPACT CT patient dosimetry

**Abstract:** We modified the Imaging Performance Assessment of CT scanners (ImPACT) to evaluate the organ doses and the effective dose based on the International Commission on Radiological Protection (ICRP) Publication110 reference male/female phantom with the Aquilion ONE vision edition scanner. To select the new CT scanner, the measurement results of the CTDI_100,c_ and CTDI_100,p_ for the 160 (Head) and the 320 (Body) mm polymethylmethacrylate phantoms, respectively, were entered on the Excel worksheet. To compute the organ doses and effective dose of the ICRP reference male/female phantom, the weighting factors reported by Zhang et al. were applied. The organ doses and the effective dose were almost identical for the ICRP reference male/female and modified ImPACT. The results of this study showed that, with the dose assessment of the ImPACT, the difference in sex influences only testes and ovaries. Because the MIRD-5 phantom represents a partially hermaphrodite adult, the phantom has the dimensions of the male reference man including testes, ovaries, and uterus but no women breasts, whereas the ICRP male/female phantom includes whole-body male and female anatomies based on high-resolution anatomical data sets. The weighting factors reported by Zhang can be used to estimate the doses of a male and a female accurately, and efficient dose assessment can be performed with the modified ImPACT.

Key words: computed tomography, ImPACT, effective dose, organ dose, ICRP

PACS number: 87.53.Bn, 87.57.Q-, 87.57.-s
